# Laparoscopic nephrectomy, ex vivo renal artery aneurysm repair, and autotransplantation for symptomatic aneurysm with thromboembolism

**DOI:** 10.1016/j.jvscit.2023.101220

**Published:** 2023-05-23

**Authors:** Lorela Weise, Xuanji Wang, Luis Fernandez, Bernadette Aulivola

**Affiliations:** Department of Surgery, Loyola University Medical Center, Maywood, IL

**Keywords:** Autotransplantation, Ex vivo repair, Renal artery aneurysm

## Abstract

Renal artery aneurysms involving segmental branches pose a technical challenge to repair. Both endovascular and open repair techniques have been described. This case illustrates the clinical presentation of a patient with a symptomatic renal artery aneurysm with thromboembolic renal infarction managed with laparoscopic nephrectomy, ex vivo aneurysm resection, renal artery reconstruction, and autotransplantation.

Renal artery aneurysms (RAAs) are rare, occurring in 0.1% to 1.3% of the general population.^1^^,^^2^ Nearly two thirds are located at the main renal artery bifurcation.^2^ The indications for repair include symptoms, rupture, size >3 cm, and aneurysms of any size in women of childbearing age or associated with refractory hypertension and renal artery stenosis.^3^ Open and endovascular techniques have been described; however, surgical reconstruction is recommended for suitable patients when multiple segmental branches are involved. Ex vivo repair with autotransplantation is preferable over nephrectomy when feasible to preserve renal function.^3^ The patient provided written informed consent for the report of her case details and imaging studies.

## Case report

**Fig 1:**
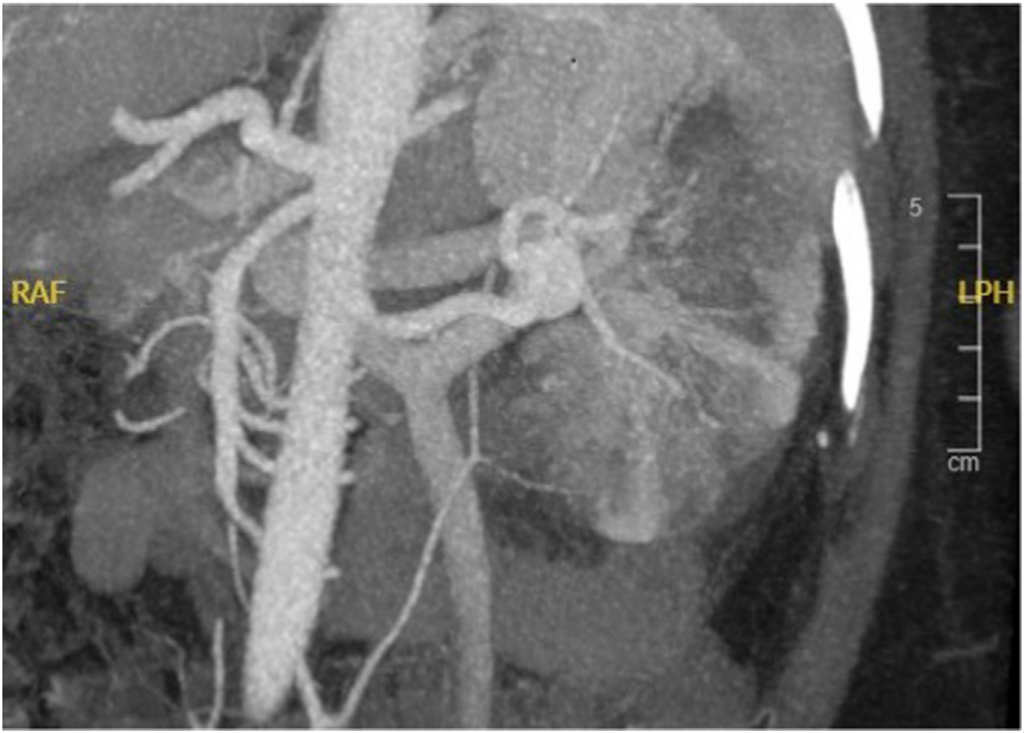
Computed tomography angiogram showing distal left renal artery aneurysm (RAA) involving superior and inferior segmental arteries and renal infarct involving mid- and lower pole of kidney.

A 44-year-old woman presented to the emergency department with acute-onset left flank pain and gross hematuria. The laboratory workup revealed a normal creatinine (0.69 mg/dL). Computed tomography angiography demonstrated a partially thrombosed, 2.0-cm, distal left RAA involving the superior and inferior segmental renal arteries. Heterogeneous hypoenhancement of the lower pole of the kidney was seen, consistent with infarction (Fig 1). Given the concern for thromboembolism, therapeutic anticoagulation was initiated. We discussed various treatment options, including endovascular stent graft repair with embolization of the lower pole segmental renal artery branch, coil embolization, open surgical reconstruction, nephrectomy, and conservative management with anticoagulation therapy. However, the need for repair of some form was emphasized. A renal perfusion/functional scan revealed 39% of renal activity attributed to the left and 61% to the right kidney. Given the goal of preserving maximal renal function, we decided to perform laparoscopic left nephrectomy, back-table aneurysm resection with renal artery reconstruction, and kidney autotransplantation.

With the patient under general anesthesia, she was placed in a right lateral decubitus position, and laparoscopic left nephrectomy was performed. The left kidney was removed via a suprapubic incision and flushed with 500 mL of cold perfusate (230 mOsm; 29 mEq/L sodium, 125 mEq/L potassium; pH, 7.4 at 20°C). The incisions were closed, the patient was repositioned supine, and the abdomen was prepared and draped while back-table renal artery reconstruction proceeded.

**Fig 2:**
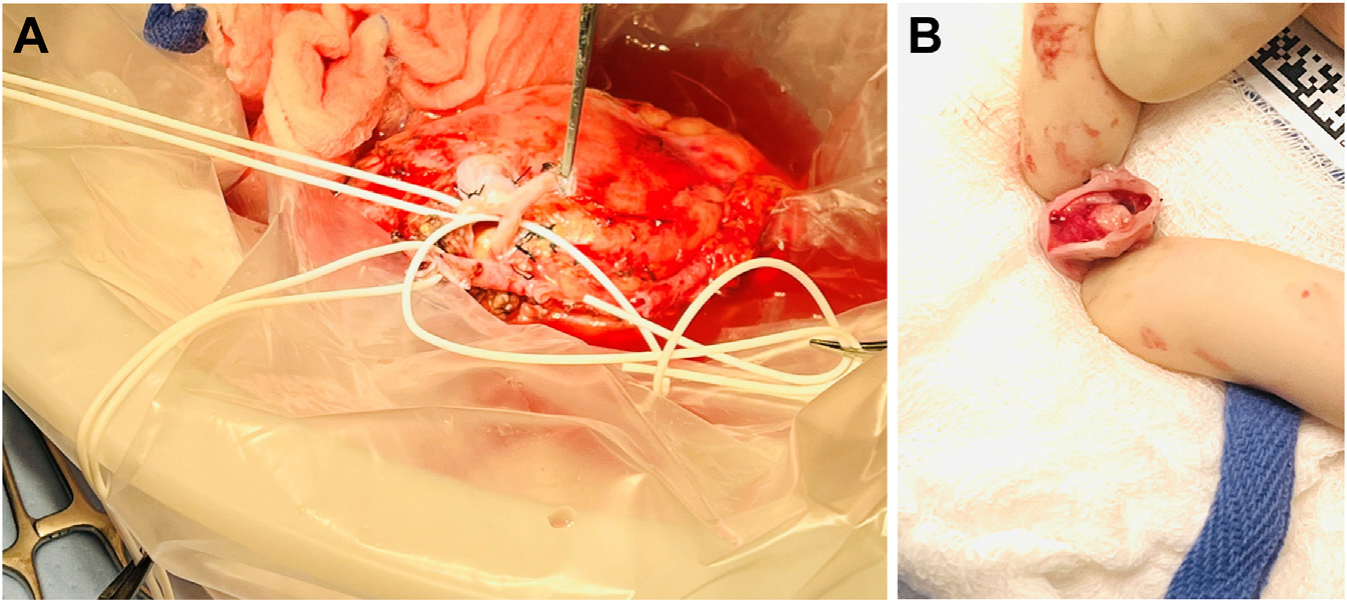
**A,** Saccular renal artery aneurysm (RAA) involving segmental branches. **B,** Intraluminal thrombus within excised RAA.

**Fig 3:**
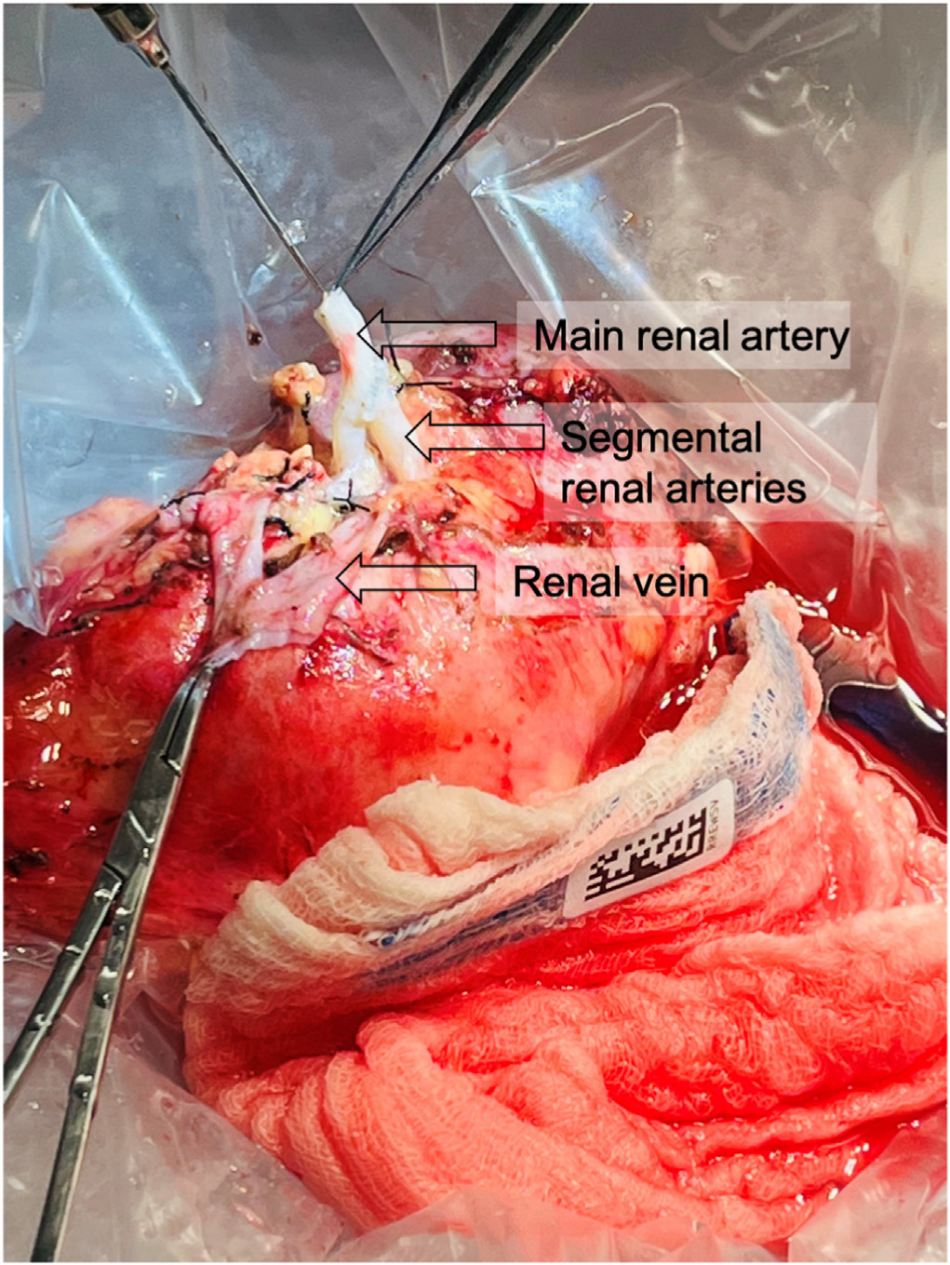
Reconstructed left renal artery with inferior segmental artery anastomosed end-to-side to superior segmental artery.

**Fig 4:**
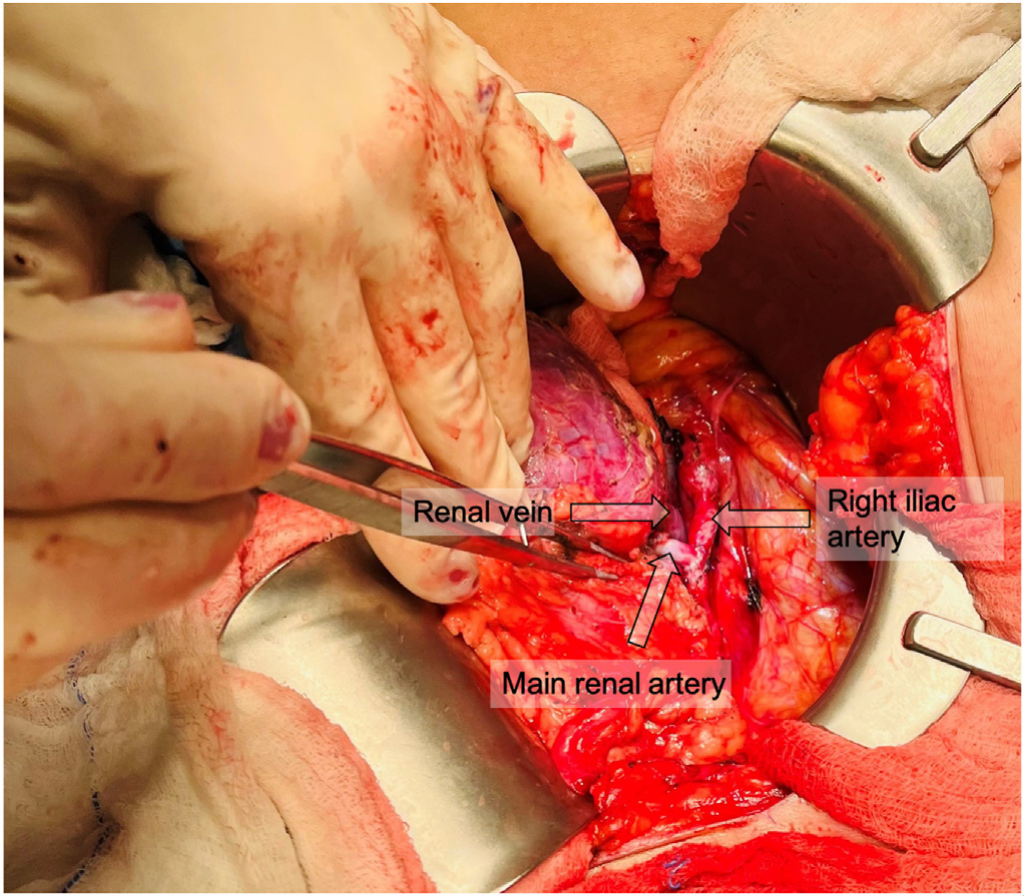
Autotransplanted left kidney anastomosed to right external iliac vessels.

The aneurysm (Fig 2, *A* and *B*) was excised, which required transection of the inferior segmental artery. The superior segmental artery was resected back to nonaneurysmal tissue. The inferior segmental artery was beveled and anastomosed end-to-side to the superior segmental artery with running 6-0 Prolene suture (Fig 3). The right external iliac artery and vein were exposed via an oblique right lower quadrant abdominal incision. The renal vein, followed by the artery, were anastomosed end-to-side to the right external iliac vessels (Fig 4). Ureteroneocystostomy was performed over a double-J ureteral stent. The renal parenchyma initially appeared pink and well-perfused but shortly after completion of the anastomoses became diffusely dusky. The renal artery pulse and Doppler signal were absent. The renal to iliac artery anastomosis was partially taken down. Thrombectomy was performed with a 3F Fogarty catheter, yielding thrombus near the anastomosis. No obvious technical issues were identified, although such were believed to be the likely cause of the thrombosis. The artery was flushed with heparinized saline, and an additional bolus of intravenous heparin was administered. The renal artery pulse and signal were restored. The kidney appeared pink and well-perfused, except for the lower pole, as expected given the prior thromboembolism-related ischemia.

Urine output remained adequate throughout the case. Low-dose heparin using a nomogram was continued postoperatively because of the initial thromboembolism and intraoperative thrombosis. The urinary catheter placed at the beginning of the case was removed on postoperative day (POD) 4. Urine cultures, sent on POD 5 given the persistent dysuria, revealed an enterococcus urinary tract infection (UTI), which was treated with intravenous antibiotics. The patient was discharged home on POD 10 with therapeutic rivaroxaban and aspirin 81 mg daily with plans for surveillance imaging at 1, 6, 12, 18, and 24 months postoperatively.

**Fig 5:**
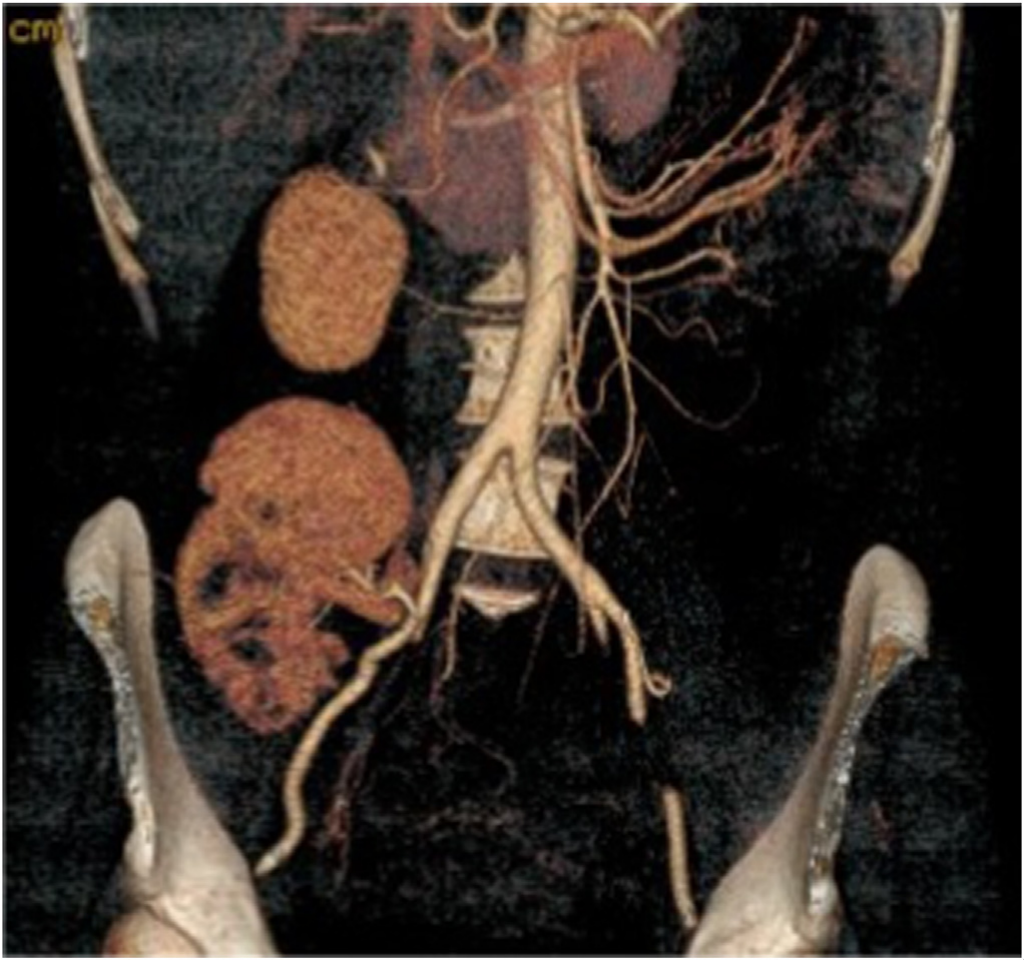
Postoperative computed tomography angiogram demonstrating autotransplanted kidney with patent renal artery and stable mid- and inferior pole infarct.

Cystoscopy and ureteral stent removal were performed 3 weeks postoperatively. A recurrent UTI, thought to be related to ureteral stent manipulation, was diagnosed at 1 month postoperatively. At that time, computed tomography angiography showed a patent autotransplanted renal artery and vein without stenosis and stable mid- and inferior pole infarct (Fig 5). The patient completed a 14-day course of antibiotics. Duplex ultrasound at 6 months postoperatively showed normal arterial and venous waveforms. Her creatinine and systolic blood pressure measurements were in the normal range throughout the follow-up period.

## Discussion

We have described the successful multidisciplinary surgical management of a symptomatic 2-cm RAA involving segmental branches. This case presents an underused, but viable, option to repair complex RAAs, with the objective of minimizing renal parenchymal ischemia and decreasing incisional morbidity. Several minor morbidities were encountered and managed, including intraoperative arterial thrombosis and postoperative UTIs. A ruptured RAA has a mortality risk as high as 55% for pregnant women and 85% for fetuses compared with 10% for non–child-bearing-age women and men. Therefore, elective repair before rupture is the ultimate goal of management. Thromboembolism, seen in the present case, occurs rarely, in only 8% to 11% of RAAs.^1^

Most RAAs occur at branch points of the main artery, with primary branches involved in 44% (average, three to four branches involved) and the main renal artery in 53%.^2^^,^^4^, ^5^, ^6^ Endovascular repair can require sacrifice of one or more branches when stent grafts are extended into branches or coils are used. One study noted an increased risk of renal infarction with endovascular management (22%) compared with open reconstruction (5%).^7^ Open surgical repair is more common for RAAs with more efferent branches.^4^

Open repair can be approached in an in vivo manner where the kidney is not dissected out of its bed, and ligation and reanastomosis of the ureter and renal vein are avoided. This is used for primary repairs that can be completed efficiently.^8^ The ex vivo approach with autotransplantation can be considered for complex RAA repairs not suitable for endovascular or in vivo repair. Complex repair often involves anastomosis of the segmental branches to the main renal artery or other segmental branches to create a common inflow channel, as was required for our patient.^8^ Less commonly, repair can require bypass with branched conduits such as a hypogastric artery or saphenous vein.

Ex vivo renal artery reconstruction facilitates renal parenchymal preservation.^4^^,^^9^^,^^10^ Duprey et al^11^ studied 67 ex vivo renal artery repairs with autotransplantation and reported no impairment of renal function, including in patients with a solitary kidney. Additionally, the patency of ex vivo repairs ranged from 82% to 99%, with in situ repairs showing a trend toward lower patency. Finally, Duprey et al^11^ reported that an ex vivo approach facilitates reconstruction for almost all aneurysmal lesions. In contrast, unplanned nephrectomy has been reported during in situ vascular reconstruction surgery. If multiple branches are involved, arterial dissection is present, or the vessels are friable, repair is more likely to involve a prolonged renal ischemia time, and ex vivo reconstruction should be pursued to minimize the warm ischemia time. The kidney can then be autotransplanted into its original bed or the iliac fossa.^8^, ^9^, ^10^

Ex vivo renal artery reconstruction can use a multidisciplinary team approach for kidney harvest and reimplantation. Laparoscopic kidney harvest has become accepted as the least morbid technique for living renal donation.^12^ It minimizes incisional morbidity and chronic pain and hastens recovery compared with a previous open technique that used a minithoracoabdominal incision extending in curvilineal fashion into the pelvis. Laparoscopic or robotic nephrectomy for ex vivo repair of RAAs has been described.^6^^,^^12^^,^^13^ Gallagher et al^6^ noted no incisional morbidity or ureteral complications, renal function remained unchanged, and arterial patency was comparable to that after open nephrectomy, with a mean hospital stay of 4 days in a series of 65 RAA repairs via laparoscopic nephrectomy and autotransplantation.

Open vs endovascular repair of RAAs has similar complication rates (12.4% vs 10.5%, respectively), with open repair having a higher incidence of cardiac and peripheral complications and a longer length of stay.^1^ Open surgical repair is associated with minor morbidity (eg, surgical site infection, UTI, ileus, urinary retention, minor renal infarct, and transient renal insufficiency) but rarely major morbidity (eg, multisystem organ failure, myocardial ischemia, renal failure requiring dialysis).^4^ Repair via either approach is safe and effective, with postoperative mortality ranging from 0% to 9.6%.^14^

## Conclusions

We describe the case of the rare presentation of a symptomatic RAA involving multiple segmental arterial branches with associated thromboembolism and partial renal infarction. The combination of laparoscopic nephrectomy, ex vivo RAA repair, and autotransplantation is a viable option for management in suitable operative candidates for preservation of renal function.
